# Should we consider assisted suicide a clinically and ethically valuable option for people with mental disorders? - Pro Speaker

**DOI:** 10.1192/j.eurpsy.2025.40

**Published:** 2025-08-26

**Authors:** T. Pollmaecher

**Affiliations:** 1 Center of Mental Health, Klinikum Ingolstadt, Ingolstadt; 2 Department of Psychiatry, Ludwig Maximilians University, Munich, Germany

## Abstract

**Abstract:**

In some European and non-European countries assisted suicide (AS), defined as supporting a person in committing suicide by providing e.g. a lethal dose of pentobarbital, is a common practice since decades. In most of these countries, AS initially was considered legitimate only, if a person has a limited expected survival time and/or suffers in an unbearable manner. Hence, AS was implicitly or explicitly not available for persons with psychiatric disorders.

Meanwhile, however, in some countries psychiatric disorders are acknowledged to cause unbearable suffering in certain cases and, in addition, a ruling of the highest court of Germany stated in 2020 that the right to seek suicide assistance might not be restricted to certain conditions or diseases, but solely tied to the competence to freely choose to end ones own life.

Because there is no indication that psychiatric disorders in general render affected person incompetent to freely choose suicide, a general exclusion of affected individuals from AS is incompatible with the biomedical principle of justice.

Clinically, however, psychiatric disorders in persons seeking AS must be seriously taken into account when a judgement has to be made on the individuals’ competence to choose freely. This kind of judgements are particularly difficult because both, the persons competence and his or her will to seek AS might be quite variable across time.

**Disclosure of Interest:**

None Declared

